# Design, Analysis, and Experiment on a Novel Stick-Slip Piezoelectric Actuator with a Lever Mechanism

**DOI:** 10.3390/mi10120863

**Published:** 2019-12-08

**Authors:** Weiqing Huang, Mengxin Sun

**Affiliations:** 1 School of Mechanical and Electrical Engineering, Guangzhou University, Guangzhou 510006, China; 2 School of Mechanical Engineering, Nanjing Institute of Technology, Nanjing 211167, China; mxsun@njit.edu.cn

**Keywords:** piezoelectric actuator, lever mechanism, analytical model, stick-slip frication

## Abstract

A piezoelectric actuator using a lever mechanism is designed, fabricated, and tested with the aim of accomplishing long-travel precision linear driving based on the stick-slip principle. The proposed actuator mainly consists of a stator, an adjustment mechanism, a preload mechanism, a base, and a linear guide. The stator design, comprising a piezoelectric stack and a lever mechanism with a long hinge used to increase the displacement of the driving foot, is described. A simplified model of the stator is created. Its design parameters are determined by an analytical model and confirmed using the finite element method. In a series of experiments, a laser displacement sensor is employed to measure the displacement responses of the actuator under the application of different driving signals. The experiment results demonstrate that the velocity of the actuator rises from 0.05 mm/s to 1.8 mm/s with the frequency increasing from 30 Hz to 150 Hz and the voltage increasing from 30 V to 150 V. It is shown that the minimum step distance of the actuator is 0.875 μm. The proposed actuator features large stroke, a simple structure, fast response, and high resolution.

## 1. Introduction

Though the piezoelectric effect was discovered more than a century ago, research on piezoelectric is still ongoing [1,2,3,4,5] and attracts attention in many areas [6,7,8,9,10]. One of the areas is actuation. Because of their advantages of simple structure, flexible design, high resolution, and low power consumption, many piezoelectric actuators have been developed [11,12,13,14]. Actuators can be classified by vibration state into the resonant type and the non-resonant type. The resonant type is the traditional one and is also called the ultrasonic motor. This type is already widely used in precision positioning, nanotechnology, and biomechanics, among other things [15,16,17,18,19]. The main drawback of this type of actuator is that the motion of the motor could be unstable as the system must work under resonance state, which is used to amplify the displacement of the driving element. Non-resonant piezoelectric actuators utilize a piezoelectric stack as the core driving element. Compared to the former type, they would produce enough deformation under the non-resonant state [20,21]. Therefore, the non-resonant actuator can operate more stably and will achieve large stroke and high resolution at the same time.

Non-resonant piezoelectric actuators can be classified into several types, among which the inchworm actuator and the inertial actuator are two important categories. The inchworm motor is a kind of bionic motor with the advantages of a strong loading capacity and high precision accuracy [22,23,24]. This type of device usually has the limitation of having a complex structure, which is difficult to assemble. In addition, the inchworm type usually requires two or three piezoelectric stacks for operation. The inertial actuator based on the inertial driving principle (stick-slip principle) often has a high pushing force [25,26,27]. In recent years, many inertia actuators with different mechanisms have been reported. Most inertia actuators are based on the friction drive principle [28,29,30]. Multilayered piezoelectric stacks (PZTs) are used to increase the amplitude of the mover displacement, and the actuators often have low operation speed because a low input voltage is used [31]. There are also reports presenting piezoelectric actuators that are designed with a flexure mechanism. The flexure mechanisms set in the actuators are not only the connecting joints to implement high precision motion, but also the amplification mechanism of the system. This type of actuator operates with the flexure mechanism to amplify the displacement of the stator per period, which increases the output speed of the mover. Significant efforts have been applied to design and analyze the flexure mechanisms in the actuators. For instance, Meng et al. proposed an approach for analyzing displacement and stiffness characteristics of flexure-based proportion compliant mechanisms based on the principle of virtual work and a rigid body model. The compliant mechanism was designed based on the numerical model and verified via finite element analysis [32]. Tian et al. utilized lever mechanisms to enlarge the working range of a five-bar compliant micro-manipulator. Numerical simulations based on linearization of trigonometric functions and a constant jacobian matrix were carried out to investigate the performance of the system. The experiment results showed that the lever mechanisms can provide the function for displacement amplification [33]. Although the above mentioned methodology can improve both the position accuracy and output displacement of the actuators, it cannot fulfill the requirements of long-stroke applications.

In previous studies, long-travel precision motions have been achieved by setting two or more perpendicular piezoelectric units. Simu et al. introduced dynamic and quasi-static motion mechanisms used in a miniature piezoceramic drive unit. The actuator consisted of six piezoelectric stacks [34]. Chen et al. proposed a piezoelectric actuators based on two groups of orthogonal structures using the friction drive principle. The experimental results indicated that the actuator can stably operate within the scope of 350 to 750 Hz when the step size is about 3.1 μm [35]. Jalili et al. proposed an actuator based on the friction drive principle with two perpendicular vibratory piezoelectric units. Experiment results showed that the maximum mean velocity was about 5.5 mm/sec, and the length of the each step was about 275 nm [36]. In these studies, the vibration of the piezoelectric stacks caused rectangular or elliptical motions at the top of the driving feet. As the driving feet are in contact with the linear guide by appropriate preload force, the micro-vibration at the top of the driving foot is transformed into the macro-linear motion of the guide. Although these actuators have high resolution and fast response, the structure and control mechanisms are complex. Dynamic errors will be caused by non-synchronized driving signals. A novel piezoelectric is proposed in this paper. Based on the mass spring damping system and the Karnopp friction model [37], theoretical analysis is carried out in the frequency domain, and the motion curve of the actuator is obtained. Simulation is carried out in ANSYS (version 18, Canonsburg, PA, USA) to verify the dynamic analysis results. Then the parameters of the flexure hinge are optimized by combining the results of numerical simulation and finite element simulation. Finally, the accuracy of the theoretical model is verified by experiments, and the characteristics of the prototype are tested.

## 2. Design of the Actuator

### 2.1. Structure of the Actuator

**Figure 1:**
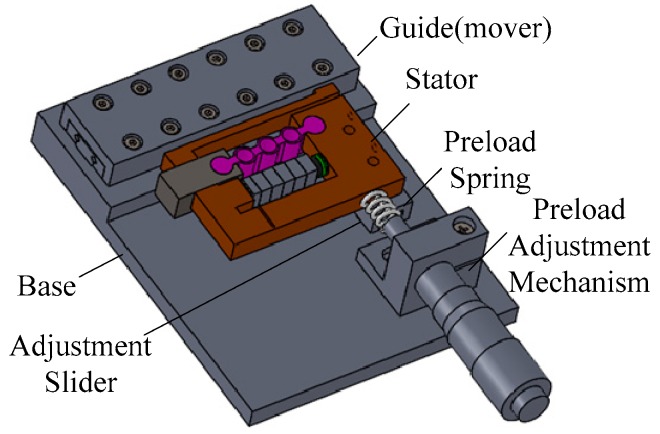
Structure of the stick-slip piezoelectric actuator.

As shown in Figure 1, a novel stick-slip piezoelectric actuator with a lever mechanism is proposed in this paper. It is comprised of a stator, an adjustment mechanism, a preload mechanism, a base, and a linear guide. As the vibration source of the actuator, a piezoelectric stack is set in the frame structure of the stator preloading by a long flexure hinge. The piezoelectric stack used is a PK4FQP1 from Thorlabs (Newton, NJ, USA). The use of a long flexure hinge has the twin functions of pre-tightening the piezoelectric stack and linking the rigid body. Since the width of the ring in the long flexure hinge is smaller than its outer radius, the hinge flexes more smoothly. Thus, the displacement produced from the piezoelectric stack will deliver to the driving foot with little error. The frame structure of the stator is designed and set on a small guide to adjust the distance between the stator and the linear guide. The preload mechanism consists of a preload spring and a preload screw mechanism to keep the stator and the linear guide in a proper contact state. When the actuator is at work, the application of an appropriate driving signal to the piezoelectric stack causes the guide to perform precision linear motion.

### 2.2. Working Principle

**Figure 2:**
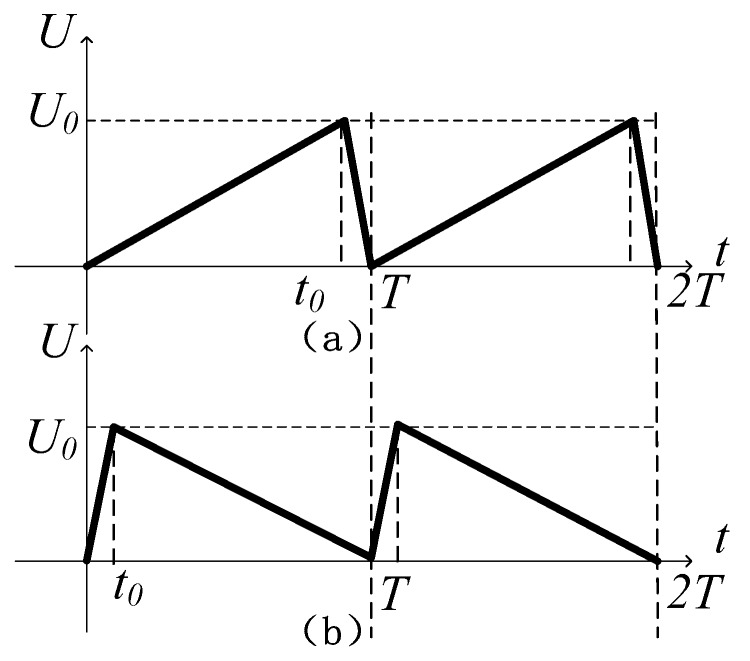
Input signal applied on the piezoelectric stack. (**a**) sawtooth signal with the voltage of slow rise and fast fall; (**b**). sawtooth signal with the voltage of fast rise and slow fall.

**Figure 3:**
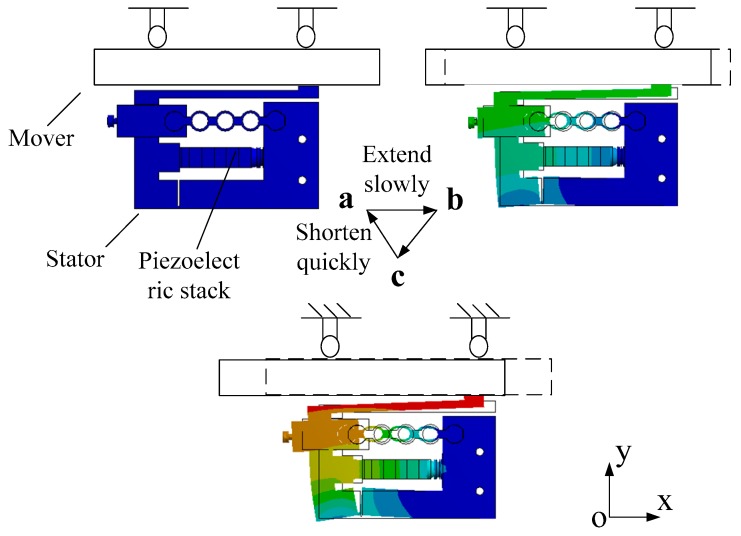
Working principle of the actuator.

The proposed stick-slip piezoelectric actuator utilizes the inertia effect to output linear motion. The input signal applied to the piezoelectric stack is shown in Figure 2. Figure 3 illustrates the working state of the stator in a full period.

The working principle of the actuator is performed by the sequence of a stick-phase and a slip-phase. Figure 2 and Figure 3 reveal that when a sawtooth wave is applied to the piezoelectric stack, the inertial skew lines can be produced at the driving foot of the stator. In one period, the proposed actuator is operated as follows: When the sawtooth wave, shown in Figure 2a, is applied to the piezoelectric stack, the piezoelectric stack extends slowly in the voltage up phase (a-b-c in Figure 3, stick-phase). Due to the driving foot being tightly clamped to the mover with the preload force, it pushes the mover to move a distance in the −*x* direction through static friction force. Then, in the voltage down phase, the piezoelectric stack quickly contracts to its initial length and drives the foot back rapidly to the initial position. In this moment, the mover moves along the −*x* direction because of its inertia (c–a in Figure 3, slip-phase). By applying the signal in Figure 2b, the movement direction of the mover will change.

### 2.3. Design and Analysis

**Figure 4:**
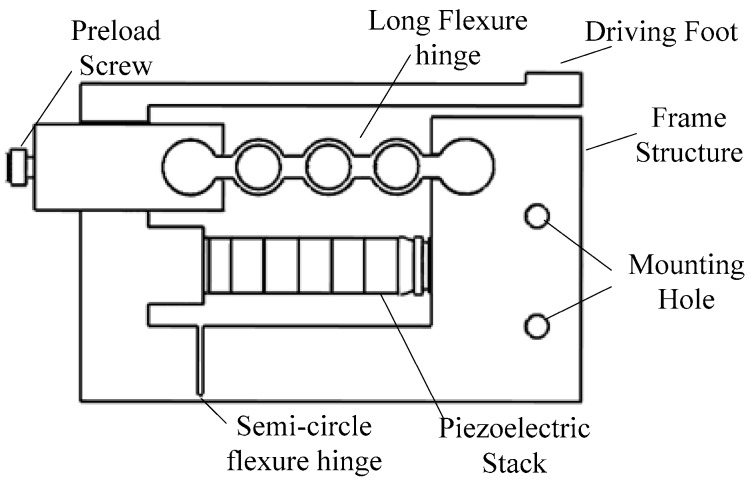
Structure of the stator.

The characteristics of the stator determine the motion performance of the actuator. To design and analyze the proposed actuator, modeling analysis is used in this paper. The structure of the stator is shown in Figure 4.

**Figure 5:**
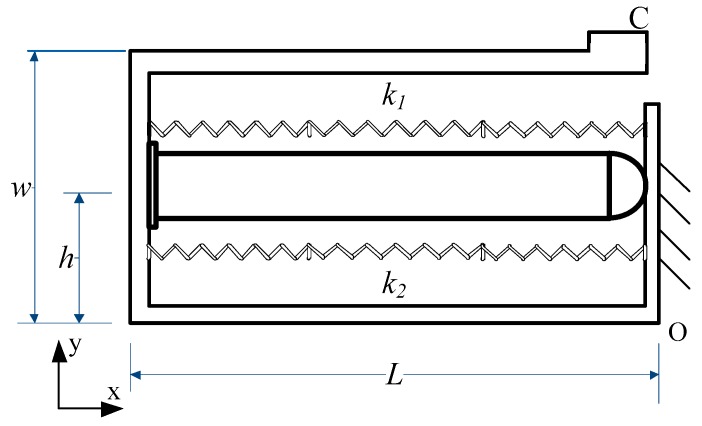
Simplified model of the stator.

Figure 5 shows the simplified model of the stator. *L* is the length of the structure, *W* is the width, *h* is the distance from the bottom of the structure to the location of the piezoelectric stack, *C* is the driving foot, and *k*_1_ and *k*_2_ represent the stiffness of the long hinge structure and the flexure hinge in the frame structure, respectively. When the stator works, the input signal applied on the piezoelectric can be written as:

(1) $$U_{p}(t)=\{\begin{array}{ll} \frac{U_{0}}{t_{0}}(t-kT), & t-kT\leq t_{0}(k=0,1,2,\ldots )\\ U_{0}-\frac{U_{0}}{T-t_{0}}(t-kT-t_{0}), & t-kT>t_{0}(k=0,1,2,\ldots ) \end{array}$$

where *U*_0_ is the maximum voltage of the input signal, *t*_0_ is the time of the peak point, and *T* is the cycle time. The output force of the piezoelectric stack is:

(2) $$F_{p}=nd_{33}U_{p}(t)k_{p}.$$

**Figure 6:**
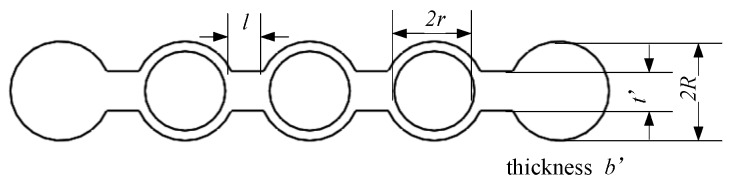
Simplified model of the long flexure hinge.

**Figure 7:**
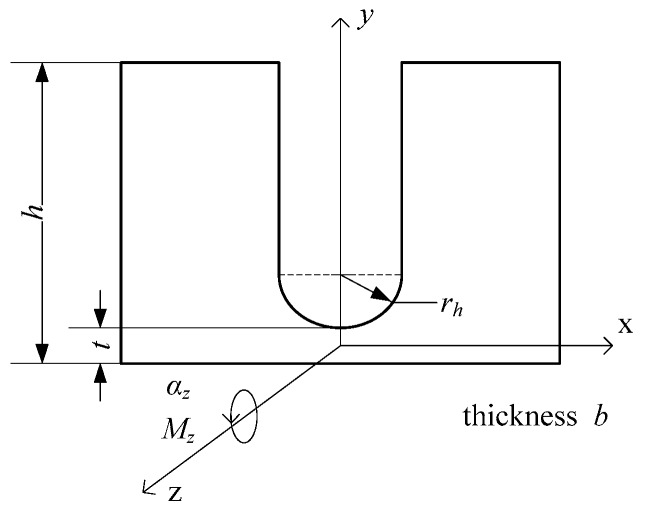
Simplified model of the semi-circular flexure hinge.

The simplified models of the long flexure hinge and the semi-circular flexure hinge are shown in Figure 6 and Figure 7. Dimension parameters of the long flexure hinge are shown in Figure 7. The long flexure hinge consists of three circular structures and four short beams. According to the knowledge of material mechanics, the equivalent tensile rigidity of the circular structure is:

(3) $$k_{11}=\frac{Eb^{\prime }{\left(R-r\right)}^{3}}{3\pi R^{3}}.$$

The equivalent tensile rigidity of the short beam is:

(4) $$k_{12}=Eb^{\prime }t^{\prime }/l.$$

It can be assumed that:

(5) $$k_{1}=\frac{k_{11}k_{12}}{3k_{12}+4k_{11}}.$$

Another flexure hinge is set in the frame structure, as shown in Figure 7. Take out the small part, whose size is *b* × *a* × *du* in the central part. The central angle is *β* and the equations to describe small part can be written as:

(6) $$a=t+r_{h}(1-\cos\beta )$$

(7) $$du=r_{h}\cos\beta d\beta .$$

When applying moment, *M_z_*, on the *z* axis and producing rotate angle, *dα_z_*, it can be expressed as:

(8) $$d\alpha_{z}=\frac{M_{z}}{EI_{z}}du$$

where *I_z_* is the inertia moment to the *z* axis:

(9) $$I_{z}=\frac{ba^{3}}{12}.$$

It can be shown that the equivalent stiffness of the semi-circular flexure hinge is [38]:

(10) $$k_{2}=\frac{M_{z}}{\alpha_{z}}=Eb/(12r_{h}\int_{-\frac{\pi }{2}}^{\frac{\pi }{2}}\frac{\cos\beta }{{\left(\frac{t}{r_{h}}+1-\cos\beta \right)}^{3}}d\beta ).$$

**Figure 8:**
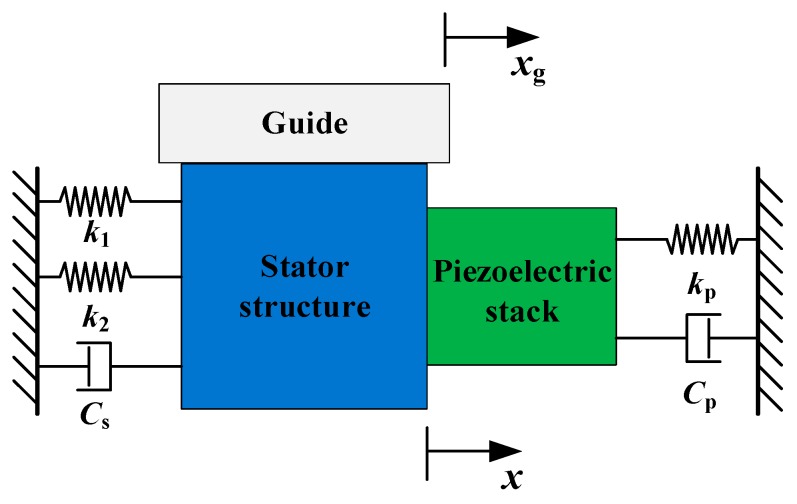
Dynamic model of the actuator.

Based on the stator analysis above, the whole movement between the stator and the mover is under consideration. Figure 8 shows the dynamic model of the actuator.

**Figure 9:**
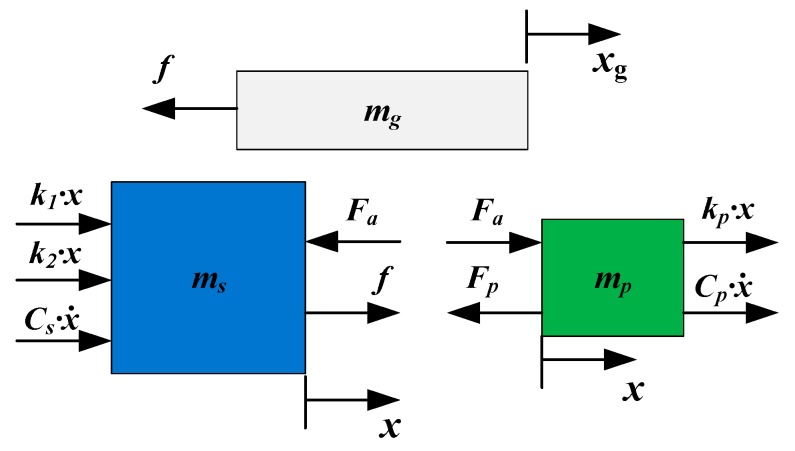
Transverse force conditions in the dynamic model.

A dynamic stiffness-damping model of the proposed actuator is established, as shown in Figure 8. The transverse force condition in the dynamic model is shown in Figure 9, where *k_p_* is the stiffness of the piezoelectric stack, *C_p_* is the damping coefficient of the piezoelectric stack, *C_s_* is the damping coefficient of the stator structure, *F_a_* is the internal force of the piezoelectric stack, the stator structure, *f*, is the friction force, and *m_s_*, *m_g_*, and *m_p_* are the mass of the stator structure, piezoelectric, and guide, respectively.

The vibration equations can be written as:

(11) $$\left[\begin{array}{c} m_{p}\\ m_{s}\\ m_{g} \end{array}\right]\left[\begin{array}{ccc} \ddot{x}(t) & \ddot{x}(t) & {\ddot{x}}_{g}(t) \end{array}\right]+\left[\begin{array}{c} C_{p}\\ C_{s}\\ 0 \end{array}\right]\left[\begin{array}{ccc} \dot{x}(t) & \dot{x}(t) & {\dot{x}}_{g}(t) \end{array}\right]+\left[\begin{array}{c} k_{p}\\ k_{1}+k_{2}\\ 0 \end{array}\right]\left[\begin{array}{ccc} x(t) & x(t) & x_{g}(t) \end{array}\right]=\left[\begin{array}{c} F_{p}-F_{a}\\ F_{a}-f\\ f \end{array}\right]$$

Then the following equation can be derived:

(12) $$(m_{p}+m_{s})\ddot{x}(t)+(C_{p}+C_{s})\dot{x}(t)+(k_{p}+k_{1}+k_{2})x(t)=F_{p}-f.$$

According to the references, the friction force, *f*, can be ignored when comparing with the output force, *F_p_* [39], and the input voltage of the piezoelectric stack can be obtained as follows:

(13) $$\frac{U_{p}(s)}{U_{0}(s)}=k_{amp}\frac{1}{RCs+1}$$

where *R* is the resistance of the driving circuit, *C* is the capacitance of the piezoelectric stack, *k_amp_* is the amplification ratio of the input voltage for the piezoelectric stack, and *U*_0_ is the initial input voltage.

The following equation for the relationship between *X*(*s*) and *U*_0_(*s*) is derived by Laplace transform:

(14) $$\frac{X(s)}{U_{0}(s)}=\frac{nd_{33}k_{p}k_{amp}}{\left(RCs+1)[(m_{p}+m_{s})s^{2}+(C_{p}+C_{s})s+(k_{p}+k_{1}+k_{2})\right]}.$$

When it comes to the lever mechanism in the stator, the rotate angle is:

(15) $$\theta =\frac{x(t)}{h}.$$

Displacement of the driving foot can be written as:

(16) $$x_{C}(t)=\frac{x(t)w}{h}$$

(17) $$y_{C}(t)=\frac{x(t)L}{h}.$$

**Figure 10:**
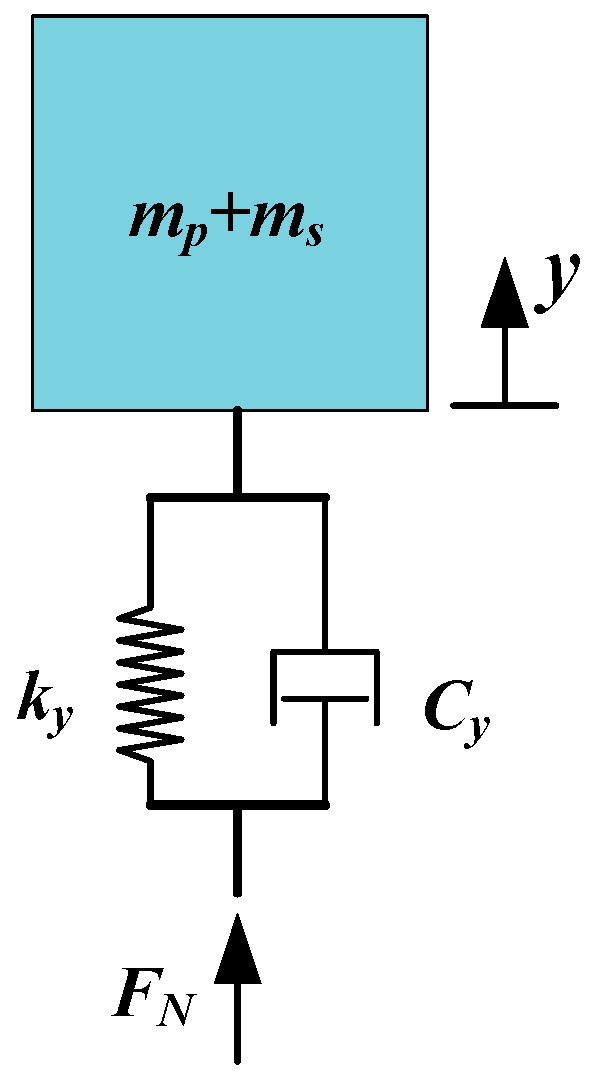
Longitudinal force condition in the dynamic model.

The longitudinal force condition in the dynamic model is shown in Figure 10, where *ky* and *Cy* are the stiffness and the damping coefficients of the stator structure in the *y* direction and *F_N_* is the preload force from the preload mechanism. The vertical force acting on the guide can be written as:

(18) $$N=F_{N}+k_{y}y(t)+C_{y}\dot{y}(t).$$

The friction force according to stick-slip can be obtained by applying the Karnopp model [37]. In this model, the friction coefficients are determined from guide velocity, *x_g_*, and the relative velocity, *v_r_*, which can be derived from:

(19) $$v_{r}=\dot{x}-{\dot{x}}_{g}.$$

The different cases of friction are described as:

(20) $$\{\begin{array}{ll} f=\frac{m_{p}+m_{s}}{m_{g}+m_{p}+m_{s}}\mu_{k}N\mathrm{sgn}({\dot{x}}_{g}), & \left|v_{r}\leq \delta v\right|(stick)\\ f=\mu_{k}N\mathrm{sgn}({\dot{x}}_{g}), & \left|v_{r}>\delta v\right|(slip) \end{array}$$

where, *μ_k_* is the kinetic friction coefficient and *δv* is the small velocity bound.

**Figure 11:**
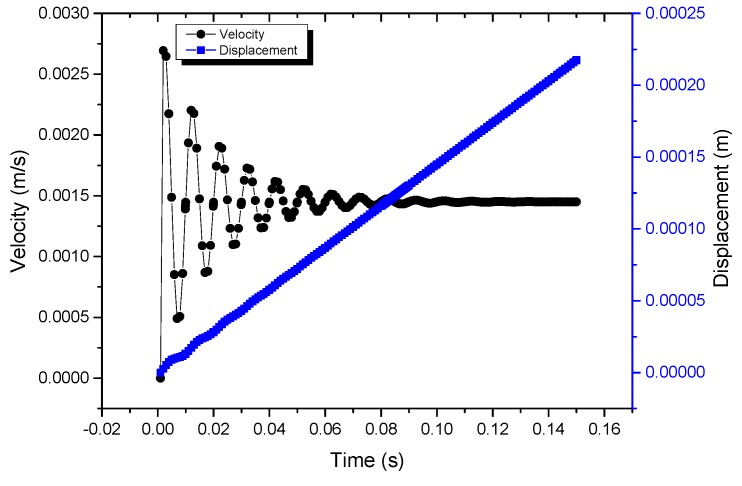
Simulation results of the actuator by MATLAB/Simulink.

The design objective of the stator is to maximize the output displacement of the contact point when it simultaneously meets the requirements of stiffness. The design parameters include the inner diameter, *r*, and width, *t*, of the long flexure hinge and the diameter, *r_h_*, of the semi-circular flexure hinge. MATLAB/Simulink (version R2016a) is used to analyze the motion characteristics, and the simulation results for the actuator are shown in Figure 11. It can be illustrated that the output velocity of the actuator is 1.45 mm/s when a sawtooth signal of 100 V and 100 Hz is applied.

**Figure 12:**
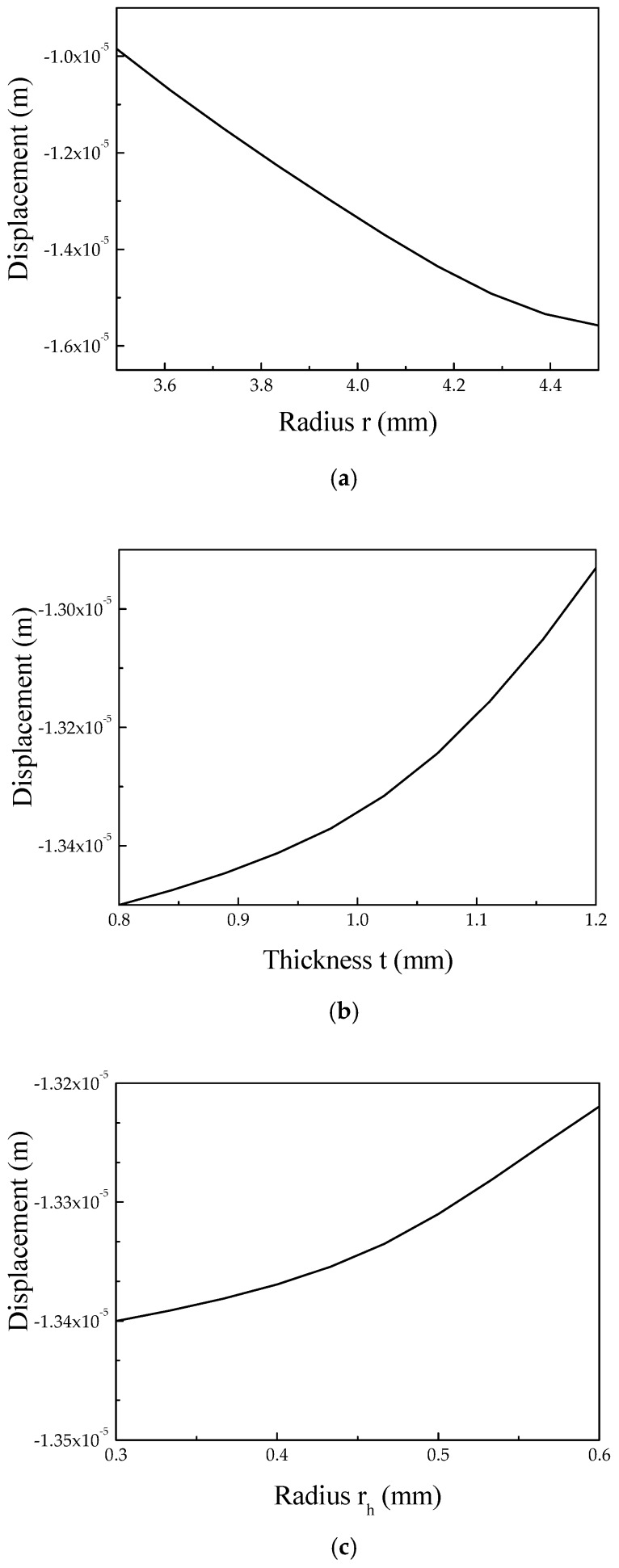
Relationships between the displacement of the driving foot versus the diameter of the long flexure hinge and semi-circular flexure hinge. (**a**) Inner diameter, *r*, and width, *t*, of the long flexure hinge versus the displacement of the driving foot. (**b**)Width, *t*, of the long flexure hinge versus the displacement of the driving foot. (**c**) Diameter, *r_h_*, of the semi-circular flexure hinge versus the displacement of the driving foot.

According to the analogue simulation, the relationships between the design parameters and the output displacement of the driving foot in the *x* direction are obtained, as shown in Figure 12.

**Figure 13:**
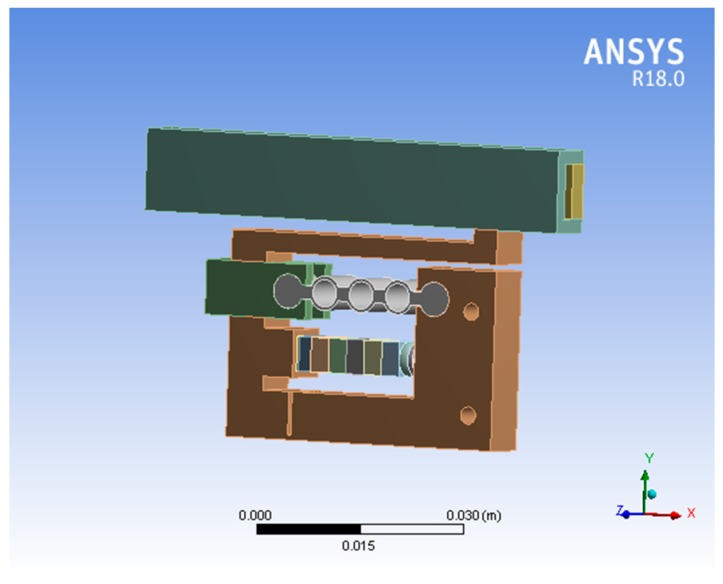
Finite element method (FEM) model.

The finite element method (FEM) of the piezoelectric actuator was performed to calculate the dynamic characteristics of the actuator when the sawtooth signal was applied. The FEM model was made up of a stator structure, a guide, and a piezoelectric stack, as shown in Figure 13. The right part of the stator was rigidly clamped. The mechanical boundary conditions of the model relate to the holding conditions used in the tests. SOLID 95 elements were used to mesh the stator parts while SOLID 98 elements were used to mesh the piezoelectric stack. The model contains 14,598 elements. The stator was made from stainless steel while the piezoelectric stack was made from piezo ceramic. Polarization was aligned in the *x* direction.

**Figure 14:**
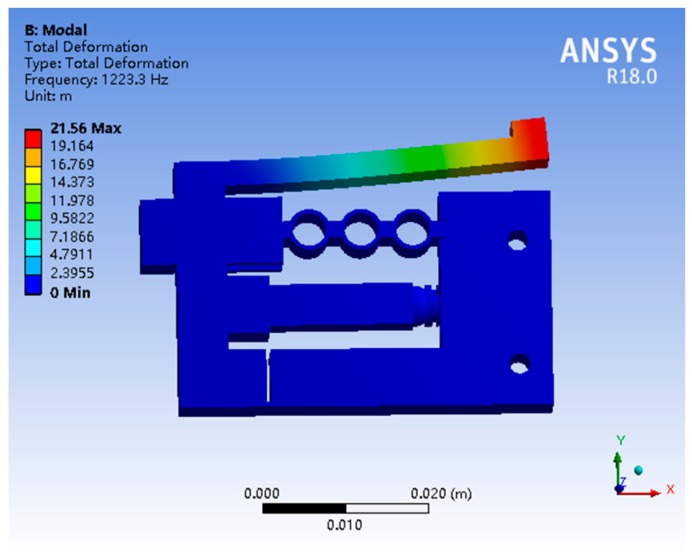
First resonant mode of the stator.

Modal frequency analysis was first performed to obtain the resonant frequency. Figure 14 shows that the first vibration mode of the stator has a 1223.3 Hz natural frequency. Since the proposed piezoelectric actuator runs on the non-resonant frequency, the working frequency of the system would be less than the resonant frequency.

**Figure 15:**
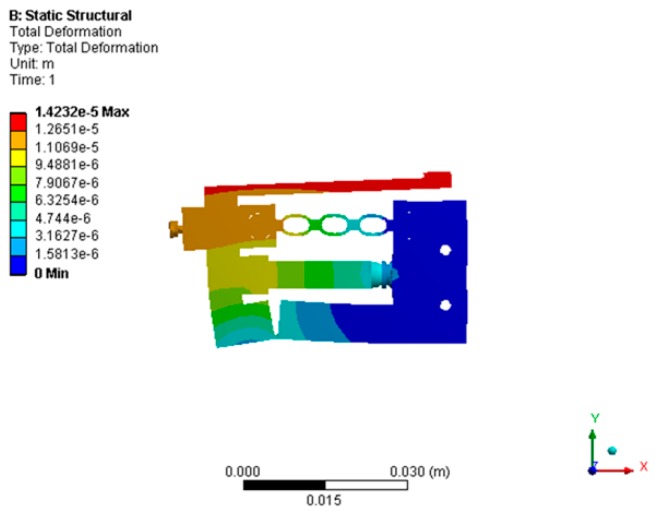
The output displacement of the driving foot.

**Figure 16:**
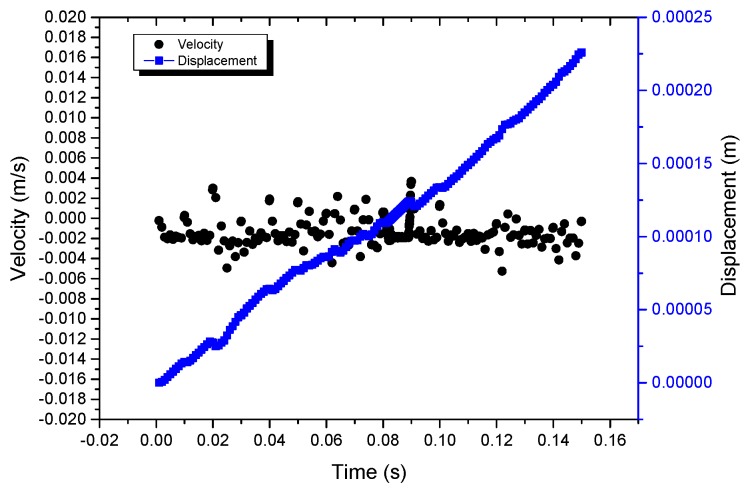
FEM dynamic analysis results.

Transient dynamic analysis was carried out next, to calculate the displacement and steady state of the actuator. When a sawtooth voltage signal of 100 Hz and 100 V was applied to the piezoelectric stack, the end of the piezoelectric stack had a displacement of about 10 μm. The output displacement of the driving foot in the *x* direction was 14.23 μm, as shown in Figure 15. At the same time, the displacement of the guide was obtained, as shown in Figure 16. It was seen that the output velocity of the actuator was 1.4 mm/s.

The FEM analysis results illustrated that the proposed piezoelectric actuator achieved high displacement and response time when a sawtooth voltage signal with low frequency was applied. Considering the simulation results, the stiffness requirement, and the size of the piezoelectric stack, the parameters were determined (*r* = 5, *t* = 1, *r_h_* = 0.5).

With the adjustment of the size of the long flexure hinge and the semi-circular flexure hinge, the resolution of the actuator can be changed to adapt to different applications. The lever amplification mechanism can increase the change rate of displacement under different voltage signals.

## 3. Experiments and Results

**Figure 17:**
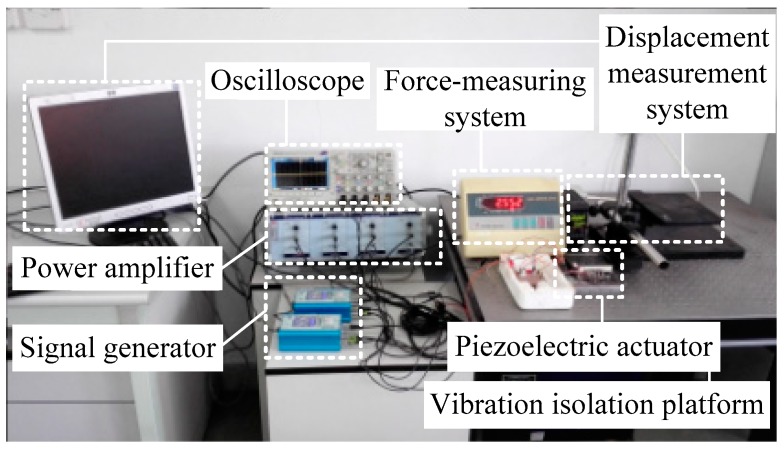
Experiment system.

**Figure 18:**
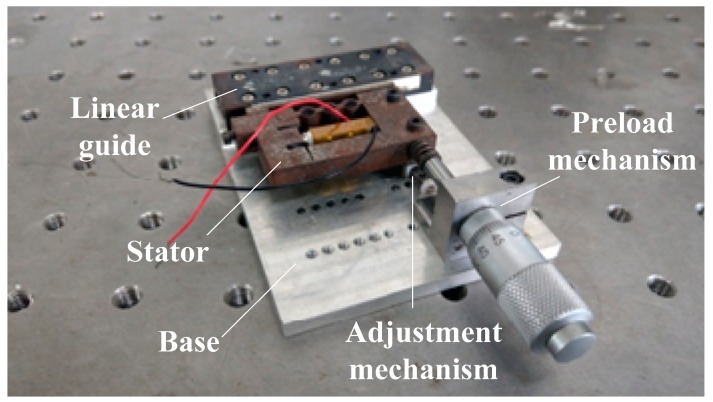
Prototype of the stick-slip piezoelectric actuator.

Figure 17 shows the established experiment system. Figure 18 shows the prototype of the stick-slip piezoelectric actuator.

### 3.1. Vibration Test of the Stator

**Figure 19:**
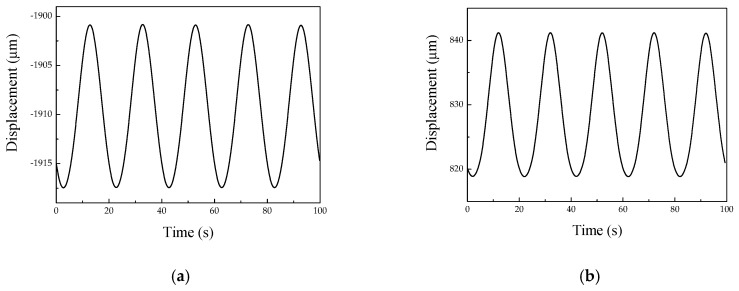
Vibration test of the driving foot. (**a**) Amplitude of the *x* direction. (**b**) Amplitude of the *y* direction.

To evaluate the effect of amplification, the displacement of the stator driving foot was tested with the help of a laser displacement sensor (KEYENCE LK-HD500). In the test, a sine wave signal with a voltage of 100 V and a frequency of 50 Hz was applied as the driving signal. Figure 19 shows the results of the displacement response measurement of the stator. According to the results, it can be seen that the average amplitude of the driving foot along the *x* direction and *y* direction was 16.5 μm and 22.3 μm, respectively, when the elongation of the piezoelectric stack was 11.5 μm.

The theoretical magnification, *m_t_*, and the experimental magnification, *m_e_*, of the *x* direction can be obtained from:

(21) $$m_{t}=\frac{14.23}{10}=1.423$$

(22) $$m_{e}=\frac{16.5}{11.5}=1.434.$$

Obviously, the experimental and theoretical results agree with each other.

### 3.2. Performance Test

**Figure 20:**
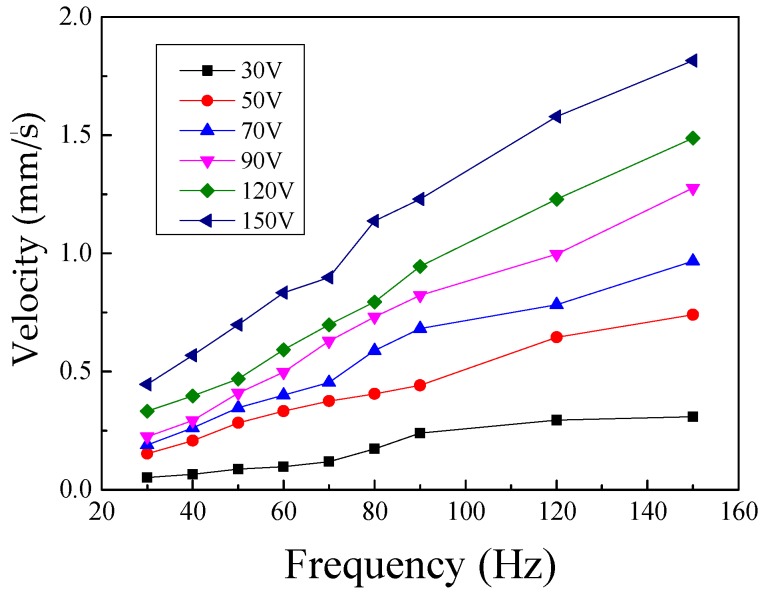
Velocity of the actuator versus the frequency by different voltage.

By utilizing a signal generator and power amplification to apply the sawtooth wave signal with different frequency and voltage on the piezoelectric stack, the relationships between the moving velocity and the driving frequency at different driving voltages were obtained and are shown in Figure 20. The results indicate that the velocity of the actuator has a linear relationship with the frequency of the driving signal under different voltages. The errors come from assembly errors and the uneven contact surface between the mover and the stator.

**Figure 21:**
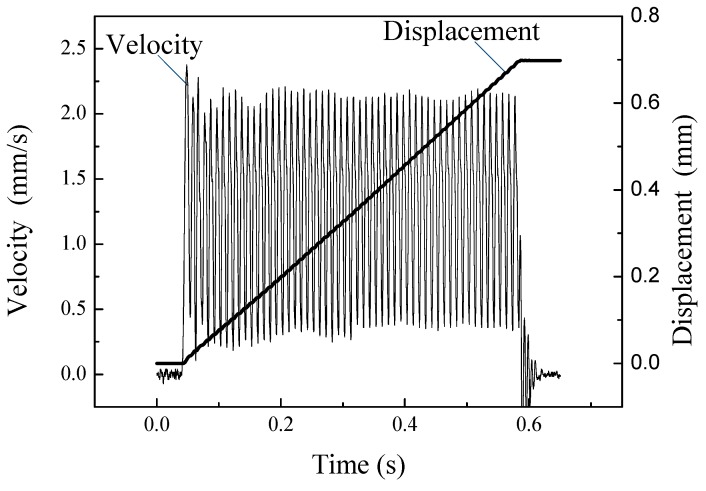
On-off operation characteristics of the actuator.

A driving signal with an input voltage of 100 V and frequency of 100 Hz was sent to the actuator, and the on-off characteristic curve of velocity and displacement was obtained, as shown in Figure 21. It illustrates that the velocity of the actuator is 1.2 mm/s in the stable state, and the response time of startup and shutdown is tens of milliseconds.

**Figure 22:**
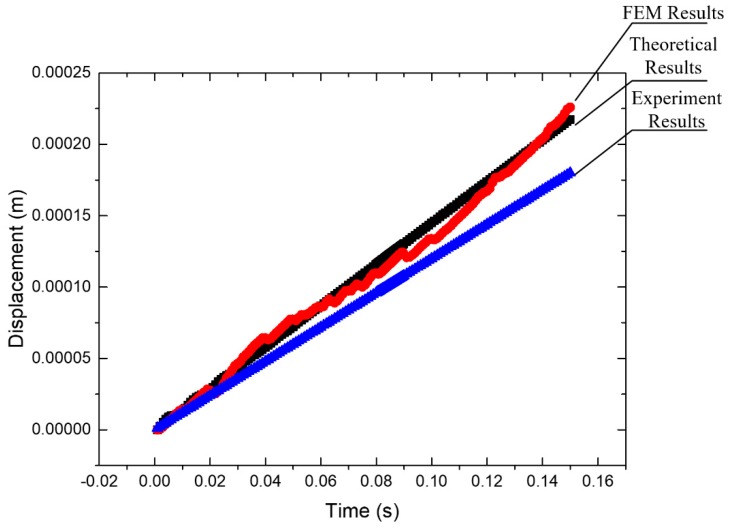
The comparison of theoretical, FEM, and the experiment results.

When the experiment results are compared to the theoretical and FEM results, the motion displacement is slightly smaller than that from the analog results, as shown in Figure 22. Due to manufacture and assembly errors, a difference would exist.

### 3.3. Resolution Test

**Figure 23:**
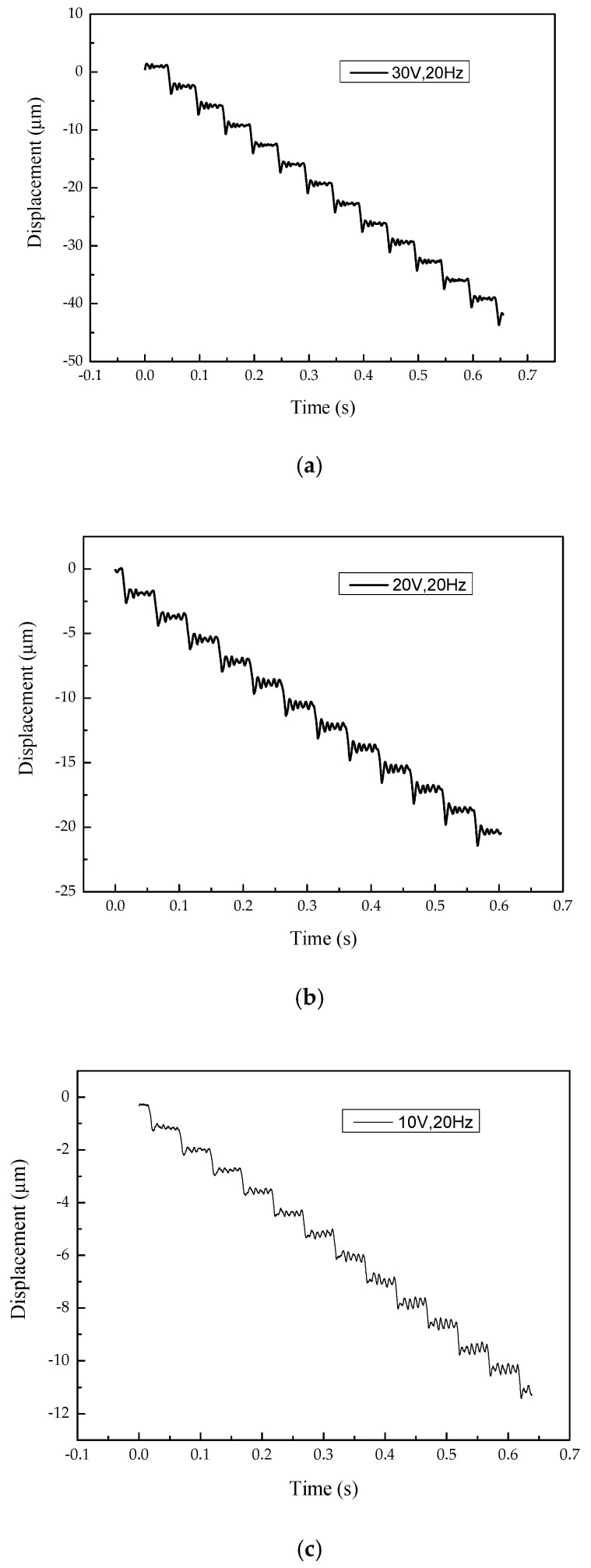
The displacement-time curve with a frequency of 20 Hz and a voltage of (**a**) 30 V, (**b**) 20 V, and (**c**) 10 V.

To measure the step distance of an actuator with a low voltage signal, a periodic pulse sawtooth wave signal was used. The experiment results are shown in Figure 23 and indicate that when the voltage of the signal is 30 V, 20 V, and 10 V, the step distance of the actuator is about 3.33 μm, 1.75 μm, and 0.875 μm, respectively. When the voltage of the input signal continues to reduce, there is no clear step distance. This is because the smaller step distance is about the same order of magnitude as the background noise. On the other hand, the movement distance of the mover in the driving phase is not much larger than the possible distance of back off in the return phase.

## 4. Conclusions

A novel stick-slip piezoelectric actuator with a lever mechanism was designed, fabricated, and tested in this research. A lever mechanism set in the stator was employed to increase the displacement of the actuator by amplifying the displacement of the driving foot. Subsequently, a long flexure hinge was used to pre-tighten the piezoelectric stack and eliminate the lateral offset error of the frame structure in the stator. Based on the modeling analysis and FEM analysis, the working principle was introduced, and the parameters of the stator were designed to meet the requirements. After the fabrication of a prototype, a vibration test of the stator and a performance test were conducted to validate the theoretical results. When a sawtooth wave with a voltage of 150 V and a frequency of 150 Hz was applied, the maximum velocity of the actuator was 1.8 mm/s. The actuator could obtained the minimum step distance of 0.875 μm from the resolution test. The results have confirmed that the design of the frame structure with a lever mechanism ensures that the actuator can undertake long-travel, fast response, and high precision linear motion.

## Nomenclature

*U*_0_	Initial voltage
*T*	Cycle time
*L*	Length of the structure
*W*	Width of the structure
*h*	Distance from bottom of the structure to the location of piezoelectric stack
*k*_1_	Stiffness of the long hinge
*k*_2_	Stiffness of the flexure hinge
*F_p_*	Output force of the piezoelectric stack
*d*_33_	Piezoelectric coefficient of the piezoelectric stack
*U_p_*	Input voltage
*k_p_*	Stiffness of the piezoelectric stack
*k*_11_	Equivalent tensile rigidity of the circle structure in the long hinge
*k*_12_	Equivalent tensile rigidity of short beam in the long hinge
*E*	Young’s modulus
*x*	Displacement of the stator
*xg*	Displacement of the guide
*Cs*	Damping coefficients of the stator structure
*Cp*	Damping coefficients of the piezoelectric stack
*Fa*	Internal force of the piezoelectric stack and the stator structure
*f*	Friction force
*ms*	Mass of the stator structure
*mg*	Mass of the guide
*mp*	Mass of the piezoelectric stack
*R*	Resistance of the driving circuit
*C*	Capacitance of the piezoelectric stack
*k_amp_*	Amplification ratio of input voltage for the piezoelectric stack
*k_y_*	Stiffness of the stator structure in the *y* direction
*C_y_*	Damping coefficients of the stator structure in the *y* direction
*F_N_*	Preload force from preload mechanism
*N*	Vertical force acting on the guide
*v_r_*	Relative velocity
*μ_k_*	Kinetic friction coefficient
*δv*	Small velocity bound
*r*	Inner diameter of the long flexure hinge
*t*	Width of the long flexure hinge
*r_h_*	Diameter of the semi-circular flexure hinge
